# B cells as modulators of HPV+ oropharyngeal cancer in a preclinical model

**DOI:** 10.3389/fonc.2023.1145724

**Published:** 2023-03-23

**Authors:** Jorge R. Galán-Ortíz, Kamila A. Andino del Valle, Abelardo A. Pérez-Rosario, Daniel L. Castañón Pereira, Jennifer Díaz-Rivera, Pamela A. Merheb-Finianos, Stephanie M. Dorta-Estremera

**Affiliations:** ^1^ Cancer Biology Division, Comprehensive Cancer Center, University of Puerto Rico, San Juan, Puerto Rico; ^2^ Microbiology and Medical Zoology Department, University of Puerto Rico, Medical Sciences Campus, San Juan, Puerto Rico; ^3^ Biology Department, University of Puerto Rico, San Juan, Puerto Rico; ^4^ Biomedical Sciences Department, San Juan Bautista School of Medicine, Caguas, Puerto Rico; ^5^ School of Medicine, Central University of the Caribbean, Bayamón, Puerto Rico

**Keywords:** B cells, HPV, PD-1 blockade, cancers, oropharyngeal

## Abstract

Among the different immune cells present within tumors, B cells also infiltrate human papillomavirus-positive (HPV+) oropharyngeal tumors. However, the role of B cells during programmed death-1 (PD-1) blockade in HPV+ oropharyngeal cancer needs to be better defined. By using the preclinical mouse model for HPV+ oropharyngeal cancer (named mEER), we characterized B cells within tumors and determined their functional role *in vivo* during PD-1 blockade. We determined that treatment naïve tongue-implanted tumors, which we have previously demonstrated to be sensitive to PD-1 blockade, contained high infiltration of CD8+ T cells and low infiltration of B cells whereas flank-implanted tumors, which are resistant to PD-1 blockade, contain a higher frequency of B cells compared to T cells. Moreover, B cell-deficient mice (µMt) and B cell-depleted mice showed a slower tumor growth rate compared to wild-type (WT) mice, and B cell deficiency increased CD8+ T cell infiltration in tumors. When we compared tongue tumor-bearing mice treated with anti-PD-1, we observed that tumors that responded to the therapy contained more T cells and B cells than the ones that did not respond. However, µMt mice treated with PD-1 blockade showed similar tumor growth rates to WT mice. Our data suggest that in untreated mice, B cells have a more pro-tumorigenic phenotype potentially affecting T cell infiltration in the tumors. In contrast, B cells are dispensable for PD-1 blockade efficacy. Mechanistic studies are needed to identify novel targets to promote the anti-tumorigenic function and/or suppress the immunosuppressive function of B cells in HPV+ oropharyngeal cancer.

## 1 Introduction

Human Papillomavirus (HPV) is the most common sexually transmitted infection in the United States and infection with oncogenic strains of HPV, such as HPV-16 and HPV-18, increases the risk of developing oropharyngeal cancer (OPC) (1, 2). HPV-related OPC is the most common site of HPV-related cancer, having passed the cervix as the most common site. Since there is no available screening test for OPC, unlike cervical cancer, the disease tends to be detected at an advanced stage (3). As the incidence of oropharyngeal cancer has increased in recent years, there is a need for the development of more effective therapies. One such therapy is *via* antibody blockade of immune checkpoint receptors, such as the programmed death receptor-1 (PD-1) (4, 5). PD-1 functions as an immune‐checkpoint regulator in T cells, and when it is ligated by PD-L1 from cancerous cells, T cell becomes anergic (6). Anti-PD-1, a monoclonal antibody, is FDA-approved to treat HNSCC and is used to block PD-1/PD-L1 interaction and promote an anti-tumor immune response. Unfortunately, the treatment is only effective in 20 – 30% of patients (5).

These poor outcomes have led to increased research regarding the tumor microenvironment (TME) mostly because tumors become infiltrated with diverse adaptative and innate immune cells that can exert pro-tumoral or anti-tumoral functions (7). Particularly, the CD8+ T cells have been a target of interest because of their potent cytotoxic abilities. Since they detect abnormal tumor antigens expressed on cancer cells and target them for destruction, their presence in the TME is correlated with a positive prognosis in patients (8, 9). However, T cell infiltration alone does not predict responsiveness to PD-1 blockade. In contrast, B cells are less studied and their role in the TME remains controversial and may exert pro-tumorigenic or anti-tumorigenic immune responses (10, 11). Some B cells with a pro-tumorigenic function, named B-regulatory cells (Bregs), tend to suppress CD8+ T cells probably through IL-10 secretion, and the anti-tumorigenic B cells may exert their functions through the formation of tertiary lymphoid structures that leads to antibody production and enhanced T cell activation (12–15).

B cells and T cells express PD-1, and therefore PD-1 blockade may also affect their function (16–19). Thus, understanding the role of B cells in the context of PD-1 blockade may be important to identify immunological mechanisms involved in PD-1 blockade resistance in HNSCC. In this study, we utilized a preclinical murine model for HPV+ oropharyngeal cancer cell line named mEER (20) to quantify lymphocytes between anti-PD-1 responding mice and non-responding mice by flow cytometry and determined whether B-cell deficiency delays tumor growth and T cell infiltration. Our goal is to elucidate the role of B cells in HPV+ oropharyngeal tumors during anti-PD-1 therapy.

## 2 Materials and methods

### 2.1 Animals

Male C57BL/6 mice (7 – 10 weeks) and B6.129S2-*Ighm^tm1Cgn^*/J were purchased from the Jackson Laboratories and were maintained in a pathogen-free environment. Only male mice were used since the mEER cell line was developed and grows better in males (20). Animal studies were pre-approved and carried out by the University of Puerto Rico Medical Sciences Campus Institutional Animal Care and Use Committee (IACUC) guidelines. Animals were anesthetized with isoflurane for tumor inoculations and blood draws, and mice were euthanized according to IACUC guidelines. DietGel 76A (ClearH2O, Westbrook, ME) was given to mice bearing tongue tumors to prevent rapid weight loss.

### 2.2 Cell line

Mouse tonsil epithelial cells expressing HPV-16 E6 and E7 and H-Ras (mEER) were a kind gift from Dr. Paola D. Vermeer (Sanford Research, Sioux Falls, SD). These cells were maintained in complete media as previously described (20), and sub-cultured at 80% confluence the day before tumor induction in mice.

### 2.3 Reagents

The tumor-infiltrating leukocytes (TILs) and tumor-draining lymph nodes were analyzed by 10-color multi-parametric flow cytometry. The antibodies we commonly use are the following: BV650 anti-CD3 (clone 17A2), BV421 anti-Granzyme B (QA18A28), BV605 anti-CD19 (6D6) FITC anti-CD11b (clone M1/70), anti-mouse CD16/32 (2.4G2, mouse Fc-block), PerCP-Cy5.5 anti-CD8 (53-6.7), BV711 ant-Gr1(clone RB6-8C5), FITC anti-PD-1 (clone 29F.1A12), PECy5 anti-CD5 (clone 53-7.3), PE anti-IgM (clone RMM-1) and PE anti-FoxP3 (clone 150D) from Biolegend (San Diego, CA). The following antibodies for *in vivo* administration were used at the concentrations shown: 250 µg dose per mouse of anti-PD-1 (RMP 1-14) from Leinco Technologies (Fenton, MO) and 100 µg dose per mouse of anti-CD20 (MB20-11) from Bio X Cell (Lebanon, NH).

### 2.4 *In vivo* tumor challenge

Mice were implanted with 5 x 10^4^ mEER cells in 50 µl PBS into the base of the tongue or 1 x 10^6^ mEER cells in 200 μl PBS subcutaneously in the flank. Subcutaneous tumors were measured in millimeters with a caliper once a week and tongue tumors were monitored by visual observations. On day 10, after tumor implantation, tongue tumor establishment was confirmed and mice treated with anti-PD-1 were divided between responders and non-responders, according to tumor disappearance or visible tumor at day 30, respectively. Mice with subcutaneous tumors were monitored closely and euthanized when a necrotic tumor was observed, the tumor measured more than 1.5 cm. Similarly, mice with tongue tumors were euthanized when mice lost 20% or more of their initial weight.

### 2.5 Immunotherapy

At day 10 after tumor challenge, mice received intraperitoneal injections of 250 µg anti-PD-1 antibody and 2 additional times at 3-day intervals (day 13 and day 16). For the anti-CD20 (MB20-11), 100 µg was injected per mouse at days 7 and 10 post-tumor implantations. Control animals were untreated.

### 2.6 Flow cytometry

For characterization of TILs, mice were euthanized between day 34 and 35 after the tumor challenge. Tongue and flank tumors were collected and digested as previously described (21). For subcutaneous flank tumors, the inguinal lymph node next to the tumor was collected and identified as a tumor-draining lymph node. For tongue tumors, the cervical lymph nodes were collected and identified as tumor-draining lymph nodes. Purified leukocytes were stained for multi-parametric flow cytometry analysis with a 10-color antibody panel. Cells were blocked with mouse Fc-block, stained with surface markers, fixed and permeabilized with the FoxP3 Fix/Perm Kit (eBioscience, Waltham, MA) followed by staining for intracellular markers. Samples were run in a 2-laser FACS Celesta flow cytometer (BD Biosciences, San Jose, CA) and analyzed using FlowJo version 10.6.2 (Flowjo LLC, Ashland, OR). The fold change was calculated by using the average cell count per lymphocyte calculated on WT mice to be analyzed as a baseline and divided by each cell count (µMT and WT).

### 2.7 B cell *in vitro* culture

Spleens from naïve C57BL/6 mice were enriched by using the mouse B cell isolation kit from Miltenyi Biotec (Bergisch Gladbach, North Rhine-Westphalia, Germany). B cells were plated at 2 x 10^6^ cells/mL in RPMI media containing 4 mM L-glutamine and 5 x 10^-5^ β-mercaptoethanol. Cells were treated with 0.1 µg/mL Resiquimod or 5 µg/mL lipopolysaccharide (LPS) with different dilutions of mEER supernatant for 24 hrs. Negative control used mEER media. After 24 hrs, cells were harvested and processed for flow cytometry.

### 2.8 Statistical analysis

All statistics were calculated using GraphPad Prism version 8 (Boston, MA). Statistical significance was determined using one-way or two-way ANOVA along with *post-hoc* correction to test differences between multiple groups or Student’s *t*-test to compare two groups. The mantel-Cox log rank test was used to compare survival curves. *P* values less than 0.05 were considered significant. Mann-Whitney test was also used to obtain the P value, (*p < 0.05).

## 3 Results

### 3.1 Inverse correlation of B cell and T cell infiltration within HPV+ oropharyngeal tumors

**Figure 1:**
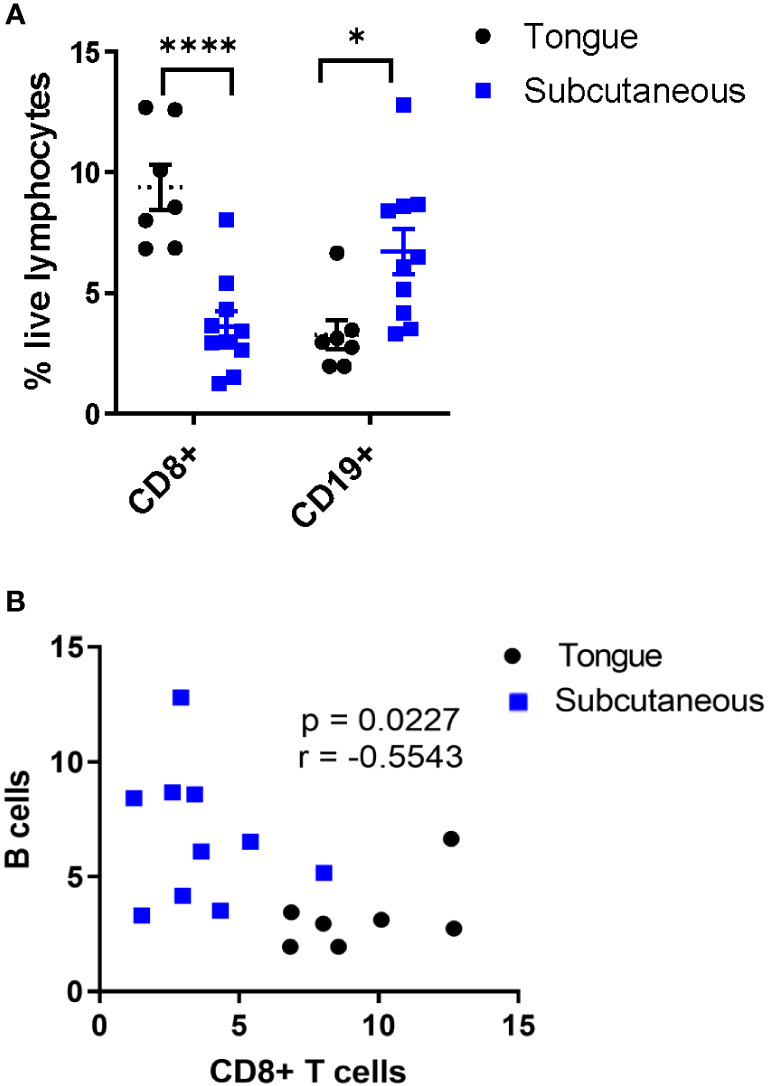
Inverse correlation between B cell and CD8+ T cell infiltration in PD-1 blockade-resistant tumors. Flow cytometry was utilized to analyze tumor-infiltrating lymphocytes present in HPV+ tumors between mice with tongue tumors (PD-1 blockade sensitive) (n = 7) or subcutaneous tumors (PD-1 blockade resistant) (n = 10). Among total live lymphocytes, CD8+ T cells and CD19+ B cells were quantified **(A)**. Significant differences between groups were determined by two-way ANOVA test ****p< 0.00001, *p < 0.01 **(A)**. The graph represents the correlation between the frequency of CD19+ B cells and CD8+ T cells in tumors (n=17) **(B)**. Results depict pooled data from 2 experiments.

We previously demonstrated that subcutaneous mEER tumors are resistant to immune checkpoint blockade whereas tongue-implanted tumors have around a 50% response rate (21), thus, we wanted to determine whether B cells differentially infiltrate these tumors. For this, frequencies of CD8+CD3+ T cells and CD19+ B cells were quantified by flow cytometry in mice bearing tumors in the tongue or subcutaneously in the flank. The data showed that tongue tumors (PD-1 blockade sensitive) contained a higher percentage of CD8+ T cells (6.8% - 12.7%) and a lower percentage of CD19+ B cells (1.95% - 6.65%) in comparison with subcutaneous tumors (PD-1 blockade resistant) which contained from 1.23% - 8.04% CD8+ T cells and from 3.31% - 12.8% CD19+ B cells (**Figure 1A**). Therefore, we found an inverse correlation between the infiltration of CD19+ B cells and CD8+ T cells on mEER HPV+ oropharyngeal tumors (**Figure 1B**). These results suggest that B cells may modulate T cell infiltration within the tumor microenvironment in this preclinical model.

**Figure 2:**
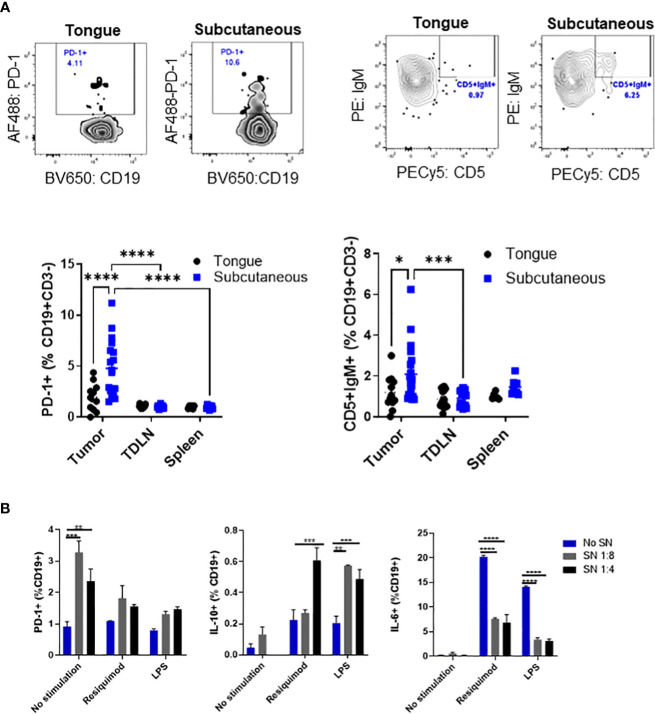
Tumor-infiltrating B cells express markers related to immunosuppression. C57BL/6 mice were injected with mEER cells in the tongue (5 x 10^4^) or subcutaneously (1 x 10^6)^ in the flank. The frequency of CD5+ and IgM+PD-1+ cells among CD19+ B cells in tumor, spleen, and tumor-draining lymph nodes were analyzed by flow cytometry after 30 days of tumor implantation. Representative flow cytometry plots are shown. Results depict pooled data from two experiments (n = 12 - 15 mice/group). Statistical analysis was done with two-way ANOVA test ****p<0.00001, ****p<0.00001, ***p<0.0001, and *p<0.01 **(A)**. After 24 hours of culture with different dilutions of tumor cell supernatant, we analyzed by flow cytometry PD-1 expression, IL-10, and IL-6 expression on stimulated mouse-enriched B cells. Statistical significance was calculated using a two-way ANOVA test: *p<0.05, **p<0.005, ****p<0.00005 (n = 2). All comparisons were made between diluted supernatant and no supernatant. This is a representative experiment of a total of 2 experiments **(B)**.

Then, we wanted to analyze immunosuppressive markers, such as CD5 and PD-1 (16, 21, 22), on B cells from different tumor sites and lymphoid organs. Representative plots of CD19+ gated cells show distinct populations for PD-1+ B cells and IgM+CD5+ B cells in subcutaneous tumors which are less distinct in tongue tumors (**Figure 2A**, top panel). We determined that subcutaneous tumors contained a higher frequency of B cells that were PD-1+ and IgM+CD5+ compared to B cells in tongue tumors (**Figure 2A**, bottom panel). The increase in the frequency of PD-1+ and IgM+CD5+ B cells was only observed in subcutaneous tumors but not tumor-draining lymph nodes or spleen on the same tumor-bearing mice.

To determine whether mEER cells may promote an immunosuppressive B cell subset, we cultured enriched B cells stimulated with different Toll-like receptor agonists with mEER cell supernatant to analyze the expression of PD-1, IL-10, and IL-6 by flow cytometry. It was observed a significantly higher percentage of PD-1 expression, as well as a higher frequency of IL-10-secreting B cells cultured with tumor cell supernatant (**Figure 2B**, first middle panels). In contrast, the percentage of IL-6 secretion on enriched B cells decreased after the addition of tumor cell supernatant (**Figure 2B**, last panel). Together, these results demonstrate that B cells, in addition to T cells, also infiltrate mEER HPV+ oropharyngeal tumors and express markers for immunosuppression, suggesting the presence of immunosuppressive B cell subsets.

### 3.2 B cell deficiency delays tumor growth of mEER HPV+ oropharyngeal tumors

**Figure 3:**
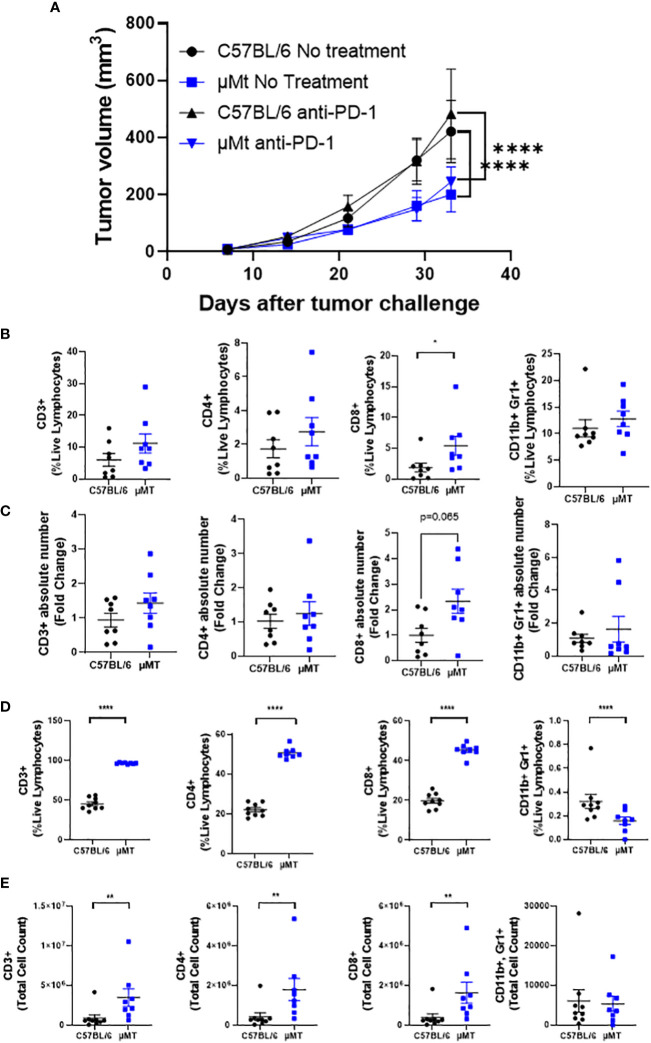
B cell deficiency delays the growth of HPV+ oropharyngeal subcutaneous tumors. C57BL/6 and µMT mice were injected subcutaneously with (1 x 10^6^) mEER tumor cells and monitored for 33 days. Anti-PD-1 (250 µg) was given intraperitoneally on days 10, 13, and 16. Tumor volume was measured with a caliper once per week and twice after the third week. Graphs represent group means ± SEM (n = 9 – 10 mice/group) **(A)**. The percentage of CD3+, CD8+, CD4+, and CD11b+ Gr1+ cells between C57BL/6 and µMT mice were quantified in subcutaneous tumors at day 33 after tumor implantation by flow cytometry **(B)**. The fold changes of the TIL counts compared to C57BL/6 were calculated **(C)**. The percentage **(D)** and cell counts **(E)** of CD3+, CD8+, CD4+, and CD11b+ Gr1+ cells between C57BL/6 and µMT mice were quantified in tumor-draining lymph nodes. A Mann-Whitney test was performed *p < 0.05, **p < 0.005, ****p < 0.00005. Results depict pooled data from 2 experiments.

To determine whether B cells modulate tumor development and the efficacy of PD-1 blockade in this preclinical model, we implanted mEER cells subcutaneously into C57BL/6 (wild-type) or µMT mice (B cell-deficient) and treated or not the mice with anti-PD-1 (250 µg) on days 10, 13, and 16. As previously published, anti-PD-1 did not affect tumor growth of mEER subcutaneous tumors (21). Interestingly, we determined that B cell-deficient mice grew tumors slower compared to wild-type mice regardless of whether the mice received treatment (**Figure 3A**). Thus, PD-1 blockade did not decrease further tumor growth in B cell-deficient mice.

Since B cell deficiency was dispensable for PD-1 blockade efficacy, we wanted to understand how B cell deficiency affected tumor growth. For this, we quantified different immune cell types in tumors and tumor-draining lymph nodes between wild-type and B-cell deficient mice. We demonstrated that B cell deficiency increased CD8+ T cell frequencies and numbers in tumors compared to wild-type mice (**Figures 3B, C**, third panel). No significant differences were observed in the frequencies and numbers of total CD3+ T cells, CD4+ T cells, and CD11b+Gr1+ myeloid-derived suppressor cells. Strikingly, B cell deficiency increased the frequency and the absolute number of total CD3+ T cells, CD4+ T cells, and CD8+ T cells in tumor-draining lymph nodes (**Figures 3D, E**). In contrast, frequencies of CD11b+Gr1+ MDSCs were decreased in B-cell-deficient mice with no significant change in the absolute number (**Figures 3D, E**, last panels).

To confirm that B cells modulate tumor growth of mEER subcutaneous tumors, we utilized an anti-CD20 antibody to deplete B cells *in vivo*. Therefore, we implanted mEER cells subcutaneously and treated mice with anti-CD20 (100 µg) on day 10 after tumor implantation. We confirmed that B cell frequency in the blood was decreased at days 22 and 29 after tumor implantation (**Supplementary Figure 1A**). We observed that tumors grew slower in mice treated with anti-CD20 compared to untreated (**Supplementary Figure 1B**). As expected, when we analyzed tumors and tumor-draining lymph nodes by flow cytometry, B cell frequencies and numbers were decreased in mice that received anti-CD20 (**Supplementary Figures 1C, D**). We observed a significant increase in CD8+ T cell frequencies in anti-CD20 treated mice (**Supplementary Figure 1C**, second panel). Probably due to a small sample size, no significant differences were observed in the other immune cell populations between untreated and anti-CD20 treated mice. Nevertheless, these results show that similar to B cell-deficient mice, B cell depletion leads to slower tumor growth and increased CD8+ T cell infiltration in this preclinical model.

**Figure 4:**
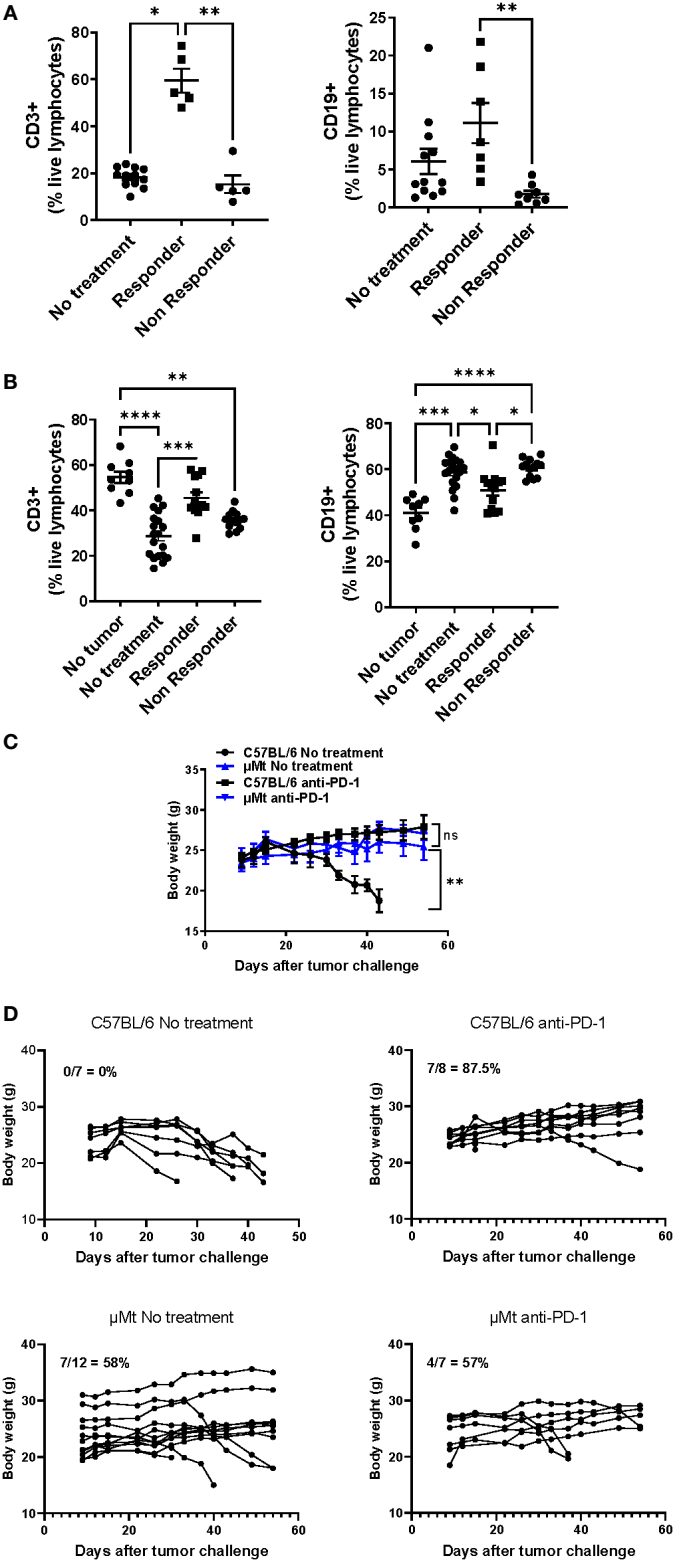
Differential infiltration of lymphocytes between PD-1 blockade responders and non-responders with HPV+ tongue tumors. C57BL/6 mice were injected in the tongue with (5 x 10^4^) mEER tumor cells and then treated with anti-PD-1 at days 10, 13, and 16. After 30 days of tumor implantation, tongues were collected and mice were classified between responders (no visible tumor) and non-responders (visible tumor); a group of mice was left untreated. CD3+ T cells and CD19+ B cells were quantified in tumors **(A)** and tumor-draining lymph nodes **(B)** by flow cytometry (n = 5 – 19). The results shown are pooled data from 3 experiments. A Mann-Whitney test was performed *p< 0.05, **p < 0.005, ***p < 0.0005, ****p < 0.00005. A group of C57BL/6 and µMT mice were injected in the tongue with (5 x 10^4^) mEER tumor cells and treated or not with anti-PD-1 on days 10, 13, and 16. Body weight was measured weekly between C57BL/6 and µMT mice treated or not with anti-PD-1. The average body weight ± SEM per group is depicted (n = 7 – 14 mice/group) **(C)**. The survival of mice was monitored over time and the percent survival per group is depicted (n = 7 – 12 mice/group) **(D)**.

Since mEER tongue tumors are sensitive to PD-1 blockade and mice may be divided between responders and non-responders, we wanted to determine whether B cells differentially infiltrate tumors that respond or not to PD-1 blockade. Interestingly, we determined that anti-PD-1 responding tumors contain higher infiltration of total CD3+ T cells (over 50% of live lymphocytes) and CD19+ B cells (over 5% of live lymphocytes) whereas non-responding tumors have significantly less lymphocyte infiltration (**Figure 4A**). The frequency of infiltration of T cells and B cells on untreated tumors was more similar to non-responding tumors. On tumor-draining lymph nodes, we observed that PD-1 blockade-responding mice showed a higher frequency of total CD3+ T cells accompanied by a lower frequency of B cells compared to non-responding mice (**Figure 4B**). Importantly, lymphocyte frequencies were similar between responders and healthy (no tumor) mice and between non-responders and treatment naïve tumor-bearing mice. These data suggest that responsiveness to PD-1 blockade correlates with T and B cell infiltration in tongue HPV+ tumors.

Lastly, we wanted to determine whether B cell deficiency affected PD-1 blockade efficacy in HPV+ tongue tumors. For this, we implanted mEER HPV+ cells in the tongue of wild-type and B-cell deficient mice (µMt). By visual examination, we confirmed tumor implantation on day 10 and then treated or not the mice with anti-PD-1 on days 10, 13, and 16. Mouse body weight was monitored. While untreated tongue-tumor-bearing mice lose weight due to tumor burden, untreated B cell-deficient mice did not lose weight. On day 60, all wild-type mice lost weight and were sacrificed due to tumor burden (**Figures 4C, D**). In contrast, tumor regression was observed in 7 out of 12 untreated B cell-deficient mice (58%) (Data not shown). Wild-type and B cell-deficient mice treated with anti-PD-1 did not show significant weight loss on average nor differences in the percent survival (**Figures 4C, D**). However, only 7 out of 8 (87.5%) anti-PD-1 treated wild-type mice had tumor regressions whereas 4 out of 7 (57%) anti-PD-1 treated B cell-deficient mice had tumors (Data not shown). Therefore, this suggests that B cell deficiency leads to impaired tumor development without affecting PD-1 blockade efficacy.

## 4 Discussion

B cell infiltration in tumors has been correlated with a favorable prognosis for patients with oropharyngeal squamous cell carcinoma (OPSCC) (10, 11). This is mostly because B cells may form tertiary lymphoid structures (TLS) that are needed for antibody class switching and somatic hypermutation (14, 15, 23). However, regulatory B cells have also been found in OPSCC and may promote an immunosuppressive microenvironment (12). In this study, we utilized the mEER HPV+ oropharyngeal cancer mouse model, a model not used before, to determine the role of B cells during HPV+ oropharyngeal cancer. In this model, tumors implanted subcutaneously in the flank do not respond to anti-PD-1 therapy whereas tumors implanted in the tongue partially respond to anti-PD-1 and this was mostly due to differential infiltration of CD8+ T cells and PD-1 expression (21). The current study demonstrated higher B cell infiltration in subcutaneous tumors than in tongue tumors, and this correlated with less CD8+ T cell infiltration. In contrast, when tongue tumor-bearing mice were treated with anti-PD-1, B cell infiltration was higher in mice that responded to the therapy. This is similar to previous reports that demonstrated that B cell infiltration correlated with responsiveness to immune checkpoint blockade in patients with melanoma and renal cell carcinoma (11, 24). Our results suggest, that in treatment naïve tumor-bearing mice, B cells may have an immunosuppressive function but anti-PD-1 therapy may promote their anti-tumor immune functions. Therefore, the mEER preclinical model where cells are implanted in the tongue may be useful to study immune responses during PD-1 blockade since their lymphocyte infiltration resembles human HNSCC cellular infiltrates during treatment (20).

Our results demonstrated that B cells were required for tumor growth since B cell deficiency and depletion of B cells with anti-CD20 antibody were correlated with delayed tumor growth. This is similar to what other preclinical cancer mouse models have found such as the EL4 thymoma, MC38 colon carcinoma, and B16 melanoma (25–27). The immunosuppressive function of B cells in these models has been attributed to IL-10 secretion and Treg recruitment (26, 28). In humans, the contrary has been observed, where the anti-CD20 antibody rituximab used alone caused rapid disease progression (29). In contrast, the combination of rituximab with the chemotherapies cisplatin and gemcitabine was feasible and safe (29). Therefore, the role of B cells in human HNSCC may play an anti-tumorigenic role. In addition to genetic differences, humans and mice have different microbiota, which are commensal bacteria that are important modulators of B cell responses (30). The human oral microbiome showed an abundance of *Porphyromonas, Fusobacteria, Veillonela, Neisseria, Streptococcus*, and *Cantonella* (31–34). In contrast, mice have shown a higher abundance of *Streptococcus, Lactobacillus, Cloacibacterium, Enterococcus*, and *Pseudomonas* (35). It is also important to recognize that the human population is heterogeneous in terms of genetics, environmental exposures, and microbial differences, and it may be possible that the immunosuppressive phenotype of B cells observed in our preclinical model represents a specific tumor stage and/or a subset of cancer patients (36). Nevertheless, identifying preclinical models that better mimic immune responses in HNSCC are greatly needed to determine the role of different immune cells in this type of cancer during treatment.

We also observed that CD8+ T cell infiltration was modulated by B cells. Differences in lymphocyte infiltration into tumors between tongue and subcutaneous tumors and after PD-1 blockade treatment may be due to differential chemokine expression. It has been reported that B cell-derived IL-35 directly inhibits CD8+ T cell infiltration in pancreatic tumors (37). Whether IL-35 may be implicated in the immunosuppressive role of B cells observed in this preclinical model needs to be investigated. In addition, CXCL13 has been implicated in B cell and T cell infiltration into tumors and in anti-tumorigenic immune responses during immune checkpoint blockade in ovarian cancer and melanoma (38, 39). It would be relevant to determine whether CXCL13 is also involved in the recruitment of B cells and T cells into oropharyngeal tumors in PD-1 blockade-responsive mice. Nonetheless, additional studies are needed to understand the mechanisms by which B cells exert their functions in HPV+ oropharyngeal cancer which may aid in the identification of novel targets to enhance the anti-tumoral responses and/or suppress the pro-tumoral effects of B cells.

## Data availability statement

The original contributions presented in the study are included in the article/**Supplementary Material**. Further inquiries can be directed to the corresponding author.

## Ethics statement

The animal study was reviewed and approved by University of Puerto Rico Medical Sciences Campus Institutional Animal Care and Use Committee.

## Author contributions

JG performed at least one experiment on all figures and contributed to the manuscript writing. KA and AP performed one replicate flow cytometry experiment from **Figures 3**, **4** and wrote sections of the manuscript, DC and JD performed one replicate experiment of tumor growth monitoring of experiment of **Figures 3**, **4**, PM performed the *in vitro* experiment of **Figure 1**, and SD provided funding, designed and supervised all experiments, and wrote and edited the manuscript. All authors contributed to the article and approved the submitted version.
