# Supplementary material for: B cells as modulators of HPV+ oropharyngeal cancer in a preclinical model
Source: Front Oncol. 2023 Mar 23;13:1145724. doi: 10.3389/fonc.2023.1145724 (PMC10076859; doi:10.3389/fonc.2023.1145724)
Supplement: Supplementary file 1 [file DataSheet_1.pdf]

Supplementary Material

1 Supplementary Figures and Tables

1.1 Supplementary Figures

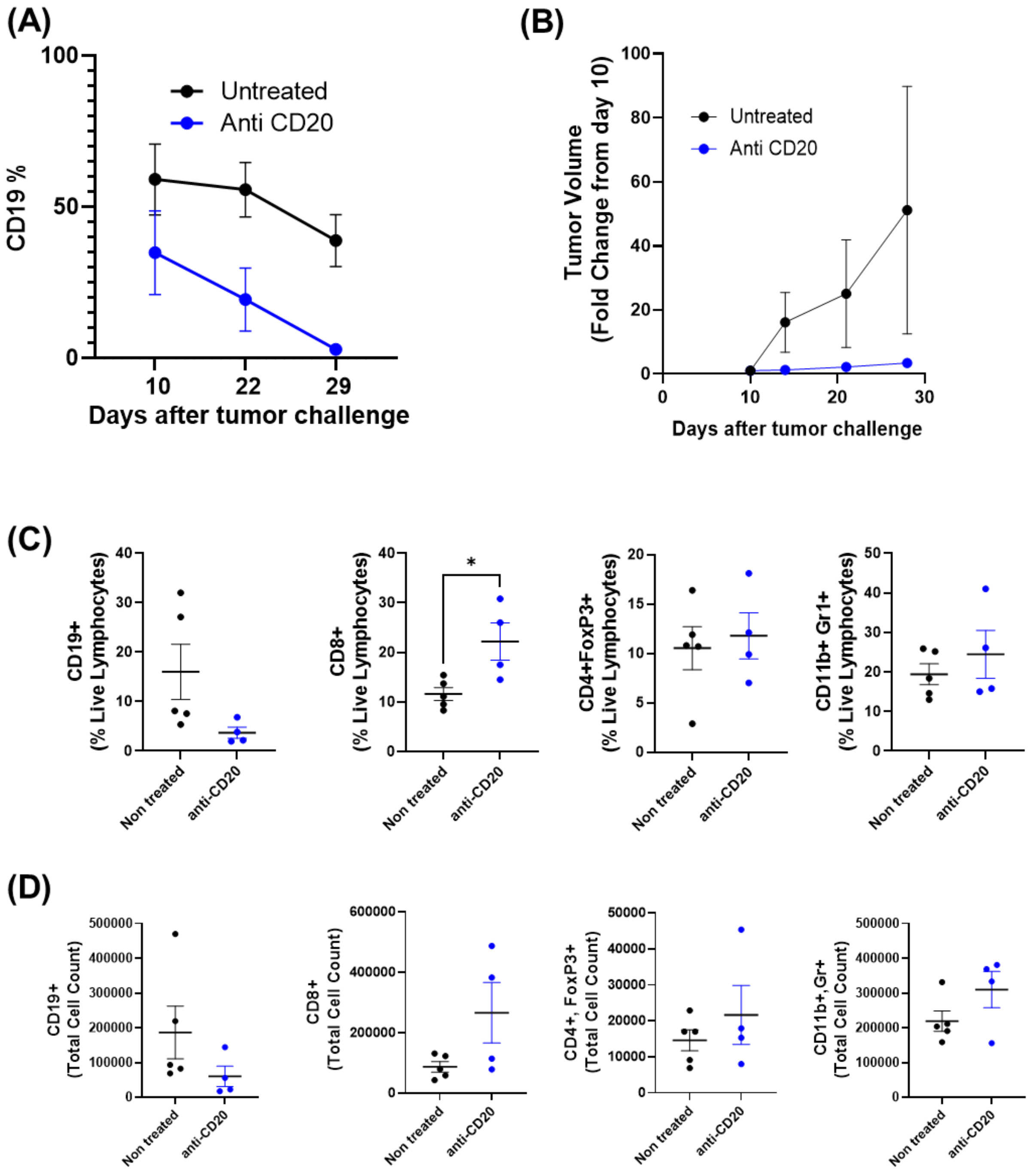

Supplementary Figure 1. B cell depletion delays tumor growth and modulates T cell infiltration in HPV+ oropharyngeal tumors. C57BL/6 mice were injected in the flank with $1x10^6$ mEER tumor cells and at day 10 after tumor implantation one group was treated with anti-CD20 (100 µg) to eliminate B cells and the other group was left untreated. Frequency of CD19+ B cells in blood at different time points was quantified between untreated and anti-CD20 treated mice by flow cytometry. Graphs represent group means ± SEM (n = 6 – 7) **(A)**. Tumor volume was monitored weekly with a caliper and blood was collected to examine the percent of CD19+ B cells by flow cytometry. Graphs represent group means ± SEM (n = 6 – 7). Statistical difference in tumor growth fold change from day 10 is represented in the two groups of mice based on an F test ***p<0.0007 **(B)**. Frequency **(C)** and total cell count **(D)** of CD19+, CD8+, CD4+FoxP3+, and CD11b+Gr1+ cells in tumors were quantified in untreated and anti-CD20 treated mice at day 36 after tumor implantation. Graphs represent individual values and group means ± SEM (n = 4 – 5). A Mann-Whitney test was performed *p < 0.05. All data was obtained from a single experiment.
